# Evaluation of long-term data on surface contamination by antineoplastic drugs in pharmacies

**DOI:** 10.1007/s00420-023-01963-y

**Published:** 2023-03-06

**Authors:** Caroline Quartucci, James P. K. Rooney, Dennis Nowak, Stefan Rakete

**Affiliations:** 1 Bavarian Health and Food Safety Authority, Institute for Occupational Health and Product Safety, Environmental Health, Munich, Germany; 2 Institute and Clinic for Occupational, Social and Environmental Medicine, University Hospital, LMU Munich, Munich, Germany; 3 Academic Unit of Neurology, Trinity Biomedical Sciences Institute, Trinity College Dublin, Dublin, Ireland

**Keywords:** Antineoplastic drugs, Surface contamination, Wipe sampling, Guidance values, Pharmacies, Occupational safety

## Abstract

**Purpose:**

The handling of antineoplastic drugs represents an occupational health risk for employees in pharmacies. To minimize exposure and to evaluate cleaning efficacy, wipe sampling was used to analyze antineoplastic drugs on surfaces. In 2009, guidance values were suggested to facilitate the interpretation of results, leading to a decrease in surface contamination. The goal of this follow-up was to evaluate the time trend of surface contamination, to identify critical antineoplastic drugs and sampling locations and to reassess guidance values.

**Methods:**

Platinum, 5-fluorouracil, cyclophosphamide, ifosfamide, gemcitabine, methotrexate, docetaxel and paclitaxel were analyzed in more than 17,000 wipe samples from 2000 to 2021. Statistical analysis was performed to describe and interpret the data.

**Results:**

Surface contaminations were generally relatively low. The median concentration for most antineoplastic drugs was below the limit of detection except for platinum (0.3 pg/cm^2^). Only platinum and 5-fluorouracil showed decreasing levels over time. Most exceedances of guidance values were observed for platinum (26.9%), cyclophosphamide (18.5%) and gemcitabine (16.6%). The most affected wipe sampling locations were isolators (24.4%), storage areas (17.6%) and laminar flow hoods (16.6%). However, areas with no direct contact to antineoplastic drugs were also frequently contaminated (8.9%).

**Conclusion:**

Overall, the surface contaminations with antineoplastic drugs continue to decrease or were generally at a low level. Therefore, we adjusted guidance values according to the available data. The identification of critical sampling locations may help pharmacies to further improve cleaning procedure and reduce the risk of occupational exposure to antineoplastic drugs.

**Supplementary Information:**

The online version contains supplementary material available at 10.1007/s00420-023-01963-y.

## Introduction

On the basis of their cytotoxic activity, antineoplastic drugs (ADs) are widely used in anticancer therapy to improve cancer survival. Some ADs are classified as carcinogenic, mutagenic, or teratogenic to humans (Connor et al. 2014; IARC 2000; Suspiro and Prista 2011). Despite the therapeutic benefits for patients, AD are recognized as a potential health risk for workers in cytotoxic drug preparation and application of cancer treatments, like pharmacists and other healthcare workers (Hon et al. 2013; Kiffmeyer et al. 2013; Odraska et al. 2014; Sessink et al. 1994; Sottani et al. 2017). The most common route for occupational exposure to AD was suggested not only to be transdermal contact with contaminated workplace surfaces or materials, but also accidental injection, inhalation or ingestion (Fransman et al. 2005; Hon et al. 2013; Kromhout et al. 2000; Lawson et al. 2019; Sessink et al. 1994). To reduce occupational exposure and achieve maximum product safety, the preparation of AD is highly regulated in hospital and private pharmacies. For example, the processing of AD is commonly performed in laminar or negative pressure boxes and in closed system drug transfer devices (Sessink et al. 2011).

For some AD, a “no observed adverse effect level” (NOAEL) cannot be derived. Therefore, the “as low as reasonably achievable” (ALARA) principle is used to minimize occupational exposure. Today, the majority of countries in Europe, the Americas, the Mideast, Far East and Australia have safe handling guidelines and practices for AD in their countries (Mathias et al. 2019). Despite these guidelines and safety measurements, studies report an ongoing surface contamination at workplaces with AD (Böhlandt and Schierl 2016; Marie et al. 2017; Segner et al. 2017) and the so affected personal remains at a potential exposure risk. Therefore, monitoring of workplace surface contamination in pharmacies and hospitals has been established as an important tool in occupational risk management (Böhlandt and Schierl 2016; Chabut et al. 2021; Palamini et al. 2020). Wipe samples are the main method to detect workplace surface contamination (Böhlandt and Schierl 2016; Colombo et al. 2017; Jeronimo et al. 2015; Schierl et al. 2009). Long-term monitoring enables pharmacies and hospitals to identify possible sources of AD release, spread and routes of exposure. It also ensures the assessment of protection procedures and supports pharmacies and hospitals to benchmark their results and to improve their procedures (Böhlandt and Schierl 2016; Schierl et al. 2016).

Our wipe sampling method for AD was first introduced in 2000 (Schmaus et al. 2002) and continuously pursued since then (Böhlandt and Schierl 2016; Schierl et al. 2009). The aim of our present study was to examine long-term data of workplace surface contamination with 5-fluorouracil (FU) and platinum (Pt, as a marker for cis-, carbo- and oxaliplatin) but also other AD from various surfaces of AD preparation areas in 181 pharmacies from 2000 to 2021 and to investigate the contamination levels over the last 21 years in more than 17,500 analyzed samples. Here, we present a follow-up of our large database of workplace surface AD concentrations and discuss whether the suggested individual guidance values (GV) introduced in 2015 are still appropriate. If not, we will propose adjusted GV based the data presented in this study.

## Methods

### Monitoring program for antineoplastic drugs

Since the early 2000s, analysis of antineoplastic drugs in surface wipe samples is offered as a service for private and public pharmacies by the Institute and Clinic for Occupational, Social and Environmental Medicine at the University Hospital, LMU Munich. The pharmacies request an individual number of samples per order for different antineoplastic drugs. A list of available methods can be found in Table 1. The provided wipe sampling material contained filters, solvents, containers as well as a detailed wipe sampling instruction. Pharmacies were asked to provide a detailed protocol including sampling area and location and to ship the samples overnight in a Styrofoam box with icepacks to keep the samples refrigerated in order to minimize potential degradation after sampling. A detailed description of the sampling locations is provided in table S1.

**Table 1:** Characteristics of the methods and analytes available the analysis of antineoplastic drugs (AD) in surface wipe samples

Method	AD	Wiping solvent	Extraction solvent	Analytical method	LOD [ng/sample]	LOD* [pg/cm^2^]
1	Total platinum	0.1% hydrochloric acid	2% hydrochloric acid	Voltammetry	0.02	0.05
2	5-fluorouracil	Methanol	Methanol	GC–MS/MS after derivatization	0.2	0.5
3	Cyclophosphamide, ifosfamide	Methanol	Ethyl acetate	GC–MS/MS after derivatization	0.2	0.5
4	Cyclophosphamide, ifosfamide, gemcitabine, methotrexate, docetaxel, paclitaxel	Water	Methanol	LC–MS/MS	0.2	0.5

*Limit of detection (LOD) using a sampling area of 400 cm^2^

### Sampling and analytical procedure

The sampling followed an established procedure (Schmaus et al. 2002). In detail, each surface was consecutively wiped with three filters (Quantitative Grade 391 Filter Papers, 90 mm diameter, Sartorius, Göttingen, Germany) moistened with an appropriate solvent (Table 1). The filters for each sampling location were collected into a single screw-cap glass container, shipped overnight to the laboratory and stored at  – 20 °C until analysis. All analytes were extracted with a suitable solvent prior to analysis (Table 1). Total Pt concentrations in wipe samples were determined by voltammetry as a marker for platinum-containing antineoplastic drugs. FU as well cyclophosphamide (CP) and ifosfamide (IF) was analyzed after derivatization by GC–MS/MS. CP and IF were also included in a LC–MS/MS multimethod along gemcitabine (GEM), methotrexate (MTX), docetaxel (DOC) and paclitaxel (PAC). Detailed information on the analytical methods is given in the supplemental information.

### Statistical analysis

First, the data were collected in tabular form using Excel, Version 15.0 (Microsoft Corporation, Redmond, USA). All results were adjusted to the size of the sampled area. Consequently, only samples with a known sampling area were included in the dataset by default. For the graphical presentation of the data, results below the limit of detection (LOD) were set to half LOD. In case of the recommended and predominant wipe sampling of 400 cm^2^, the resulting area-adjusted LOD is 0.05 pg/cm^2^ for Pt and 0.5 pg/cm^2^ for all other AD. Results were grouped into different locations based on the sampling protocol of the pharmacies. Guidance values (GV) used to assess the severity of contamination are given in Table 2. Levels between GV-I and GV-II are considered an “intermediate” contamination, above GV-II a “high” contamination. For FU and Pt, GV proposed by Schierl et al. were used (Schierl et al. 2009). For all other AD, previously available data was insufficient to establish statistically derived GV. Therefore, preliminary GV were used to evaluate the contamination. To evaluate the relationship between the different outcomes and factors, multivariable linear regression models with each chemical as outcome were built in turn, and each included type (public vs private), location (10 levels) and year as fixed effects were built, with an additional random effect by pharmacy. Measurements taken between 2015 and 2021 were included in these models. Descriptive statistics, multivariate linear regression models and visualization were performed using R Statistical Software (version 4.0.0).

**Table 2:** Guidance values (GV) for antineoplastic drugs (AD) used for the evaluation of the wipe sampling data

AD	GV-I in pg/cm^2^ “intermediate” contamination	GV-II in pg/cm^2^ “high” contamination
Pt*	0.6	4
FU*	5.0	30
CP	1.0	5
IF	1.0	5
GEM	1.0	5
MTX	2.0	10
DOC	3.0	15
PAC	3.0	15

^*^GV proposed bySchierl et al. (2009)

## Results

Between 2000 and 2021, 17,693 wipe samples with a known sampling area were taken in 126 public and 55 private pharmacies in Germany and Austria. Due to the use of multimethods, one wipe sample may yield multiple individual AD results. Consequently, the 17,693 wipe samples produced 29,431 individual AD results that were used for statistical analysis. The majority of pharmacies repeatedly performed wipe sampling. The number of participations per pharmacy ranged from 1 to 21 (median: 4; geometric mean: 3.8). The frequency of follow-ups is highly variable. For example, some pharmacies follow-up annually, some every 2 or 3 years. Some pharmacies stopped sampling after participating a couple time. Furthermore, some pharmacies participated for 21 years whereas other just recently conducted wipe sampling for the first time.

**Fig. 1:**
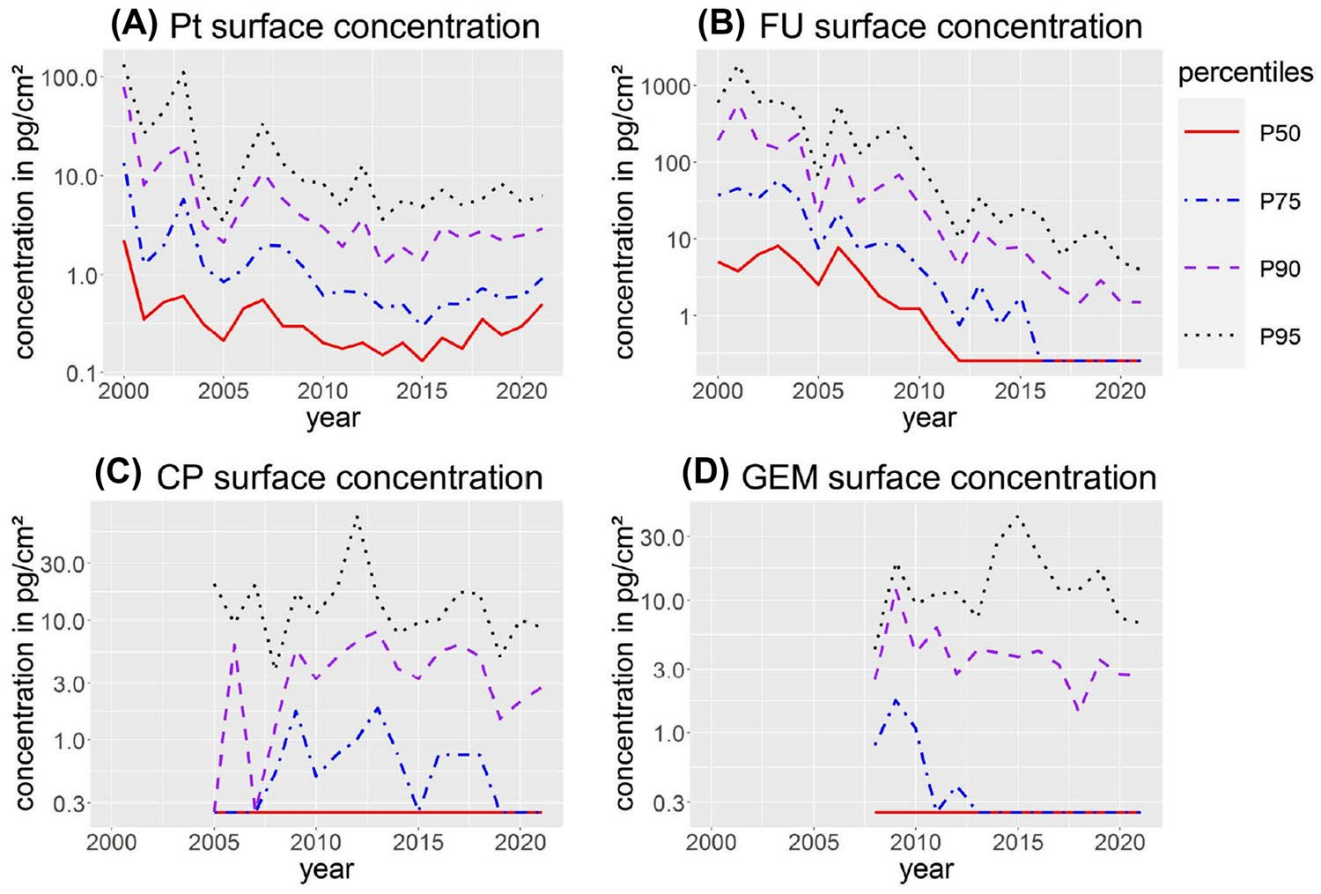
Development of selected percentiles (P50, P75, P90, and P95) of surface contaminations with platinum (**A**, Pt), 5-fluorouracil (**B**, FU), cyclophosphamide (**C**, CP) and gemcitabine (**D**, GEM) between 2000 and 2021

A descriptive statistical analysis of the results can be found in Table 2. With the exemption of Pt, the majority of results were below the limit of detection and a non-parametric distribution shifted to lower concentrations was observed for all ADs. In Fig. 1, selected quantiles (P50, P75, P90 and P95) Pt, FU, CP and GEM and their development over time are shown. For Pt, concentrations decreased until 2015 and slightly increased thereafter. In contrast, FU levels decreased until 2021, with P50 being below the LOD since 2012 and P75 since 2016. For CP and GEM, only a slight decrease of P90 and P95 was observed. For GEM, P75 stayed below the LOD since 2013. For CP, P75 varies between the LOD and concentrations lower than 2 pg/cm^2^. For IF, MTX, DOC and PAC, P50 and P75 were below the LOD throughout the entire period (Figure S1). Furthermore, the P90 for MTX, DOC and PAC were below the LOD, too. To evaluate if experience led to decreasing surface contamination, the results for Pt, FU, CP and GEM were stratified for times participated in the wipe sampling program (Figure S2). For FU, levels were highest in pharmacies that participated only once, twice or thrice and median concentrations dropped with four or more participations. In contrast, an apparent effect of times of participated was not noticed for the other Pt, CP and GEM.

**Fig. 2:**
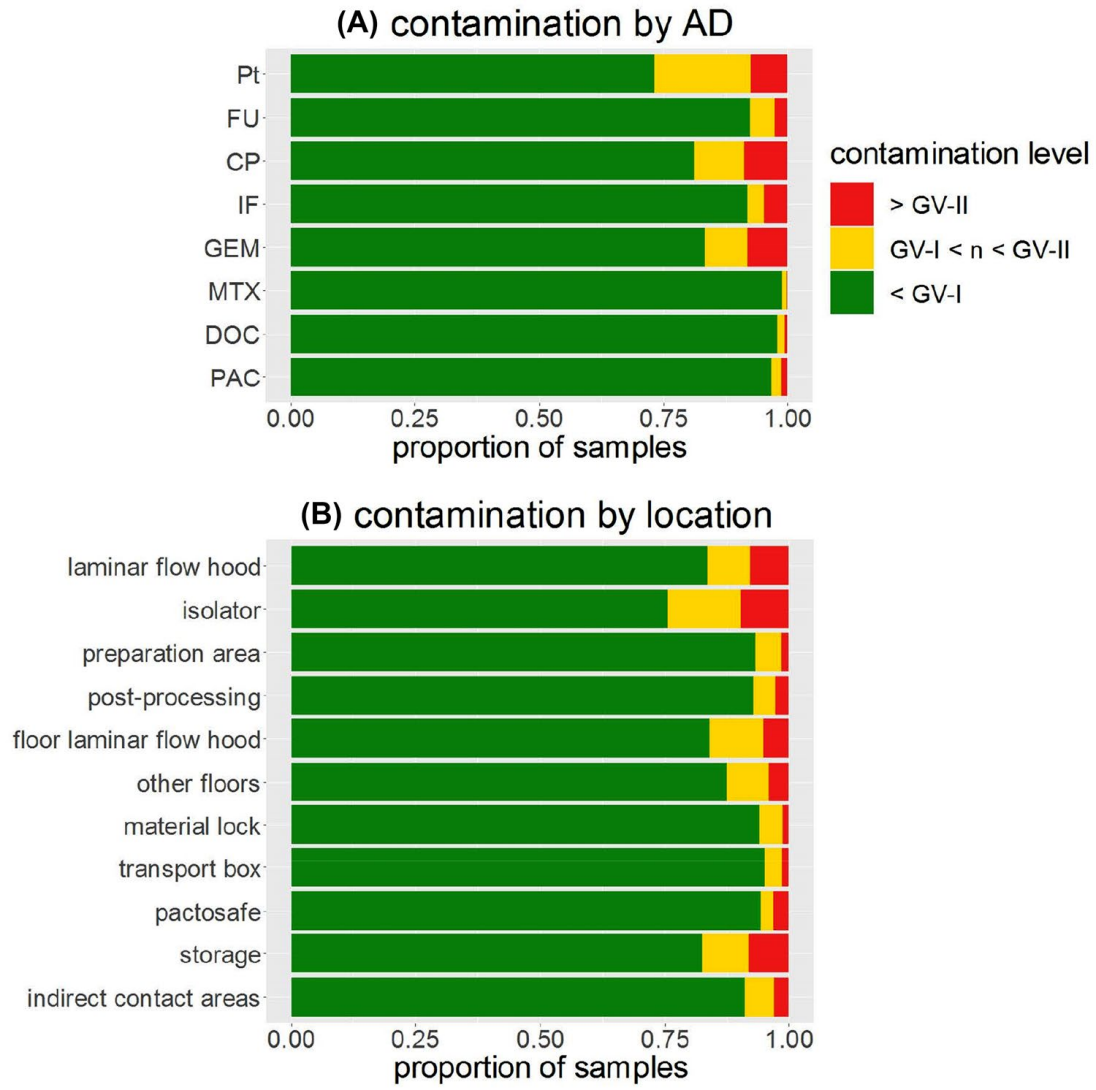
Exceedances of individual guidance values (GV, see also Table 3) for antineoplastic drugs (AD, **A**, *Pt* platinum, *FU* 5-fluorouracil, *CP* cyclophosphamide, *IF* ifosfamide, *GEM* gemcitabine, *MTX* methotrexate, *DOC* docetaxel, *PAC* paclitaxel) and sampling locations (**B**, all analytes) from 2015 to 2021. GV-I refers to an “intermediate” contamination and GV-II to a “high” contamination

By using P50 and P75, Schierl et al. proposed guidance values (GV) for Pt and FU based on the data prior to 2009 to assess the severity of contamination (Table 2) (Schierl et al. 2009). For the other AD, the available data at that time were limited and preliminary GV were used. In Fig. 2A, the percentage of exceedances of GV for each AD from 2015 to 2021 are shown. The detailed numbers of samples exceeding GV can be found in Table S2. In summary, 7.0% of all samples exceeded the GV-I, which is defined as an intermediate contamination, and 4.3% even exceeding GV-II, indicating a high contamination. However, the exceedances of individual AD varied widely. Pt showed the highest percentage of total exceedances (26.9%), followed by CP (18.5%), GEM (16.6%), IF (7.9%) and FU (7.5%). A relatively low number of total exceedances were observed for MTX (1.0%), DOC (2.0%) and PAC (3.2%).

**Table 3:** Descriptive statistics of individual wipe sampling results for each antineoplastic drug (AD) between 2000 and 2021

AD	*n*	*n* < LOD (%)	Concentration of AD in wipe samples in pg/cm^2^										
			Mean	GM	Min	P5	P10	P25	P50	P75	P90	P95	Max
Pt	4983	0 (0)	42.48	0.37	0.006	0.05	0.05	0.13	0.3	0.8	3.9	11.1	152,500
FU	6106	3554 (58.2)	434.70	0.96	< LOD	< LOD	< LOD	< LOD	< LOD	2.5	18.0	75.1	1,725,000
CP	3357	2446 (72.9)	13.65	0.52	< LOD	< LOD	< LOD	< LOD	< LOD	0.5	4.5	14.5	4228
IF	3365	2912 (86.5)	9.30	0.36	< LOD	< LOD	< LOD	< LOD	< LOD	< LOD	0.9	5.3	3960
MTX	2874	2795 (97.3)	0.36	0.26	< LOD	< LOD	< LOD	< LOD	< LOD	< LOD	< LOD	< LOD	53
GEM	2975	2252 (75.7)	18.00	0.50	< LOD	< LOD	< LOD	< LOD	< LOD	< LOD	4.1	13.8	15,450
DOC	2875	2752 (95.7)	16.47	0.28	< LOD	< LOD	< LOD	< LOD	< LOD	< LOD	< LOD	< LOD	22,500
PAC	2896	2721 (94.0)	2.02	0.29	< LOD	< LOD	< LOD	< LOD	< LOD	< LOD	< LOD	0.8	1500
Total	29,431	19,432 (66.0)											

*LOD* limit of detection, *GM* geometric mean, *P* percentile

The data were also stratified for different sampling locations. In Fig. 2B, the percentage of individual sampling locations exceeding the GV between 2015 and 2021 is shown. The detailed numbers of samples exceeding GV in specific locations can be found in Table S3. Among all locations, surfaces in the laminar flow hood were most frequently sampled (19.1% of all samples) and also showed the highest number of exceedances (39.1% of all samples exceeding the GV). However, if the number of samples above the GV is related to the total number of samples at this location, the isolator is the most commonly affected location (24.4%). In contrast, only 16.6% of the samples in laminar flow hoods exceeded the GV. Similar percentages were observed for floors in front of laminar flow hoods (16.0%) and in storage areas (17.6%). The lowest relative exceedances were observed for transport boxes (4.9%), pactosafes (5.6%) and material locks (5.9%).

**Fig. 3:**
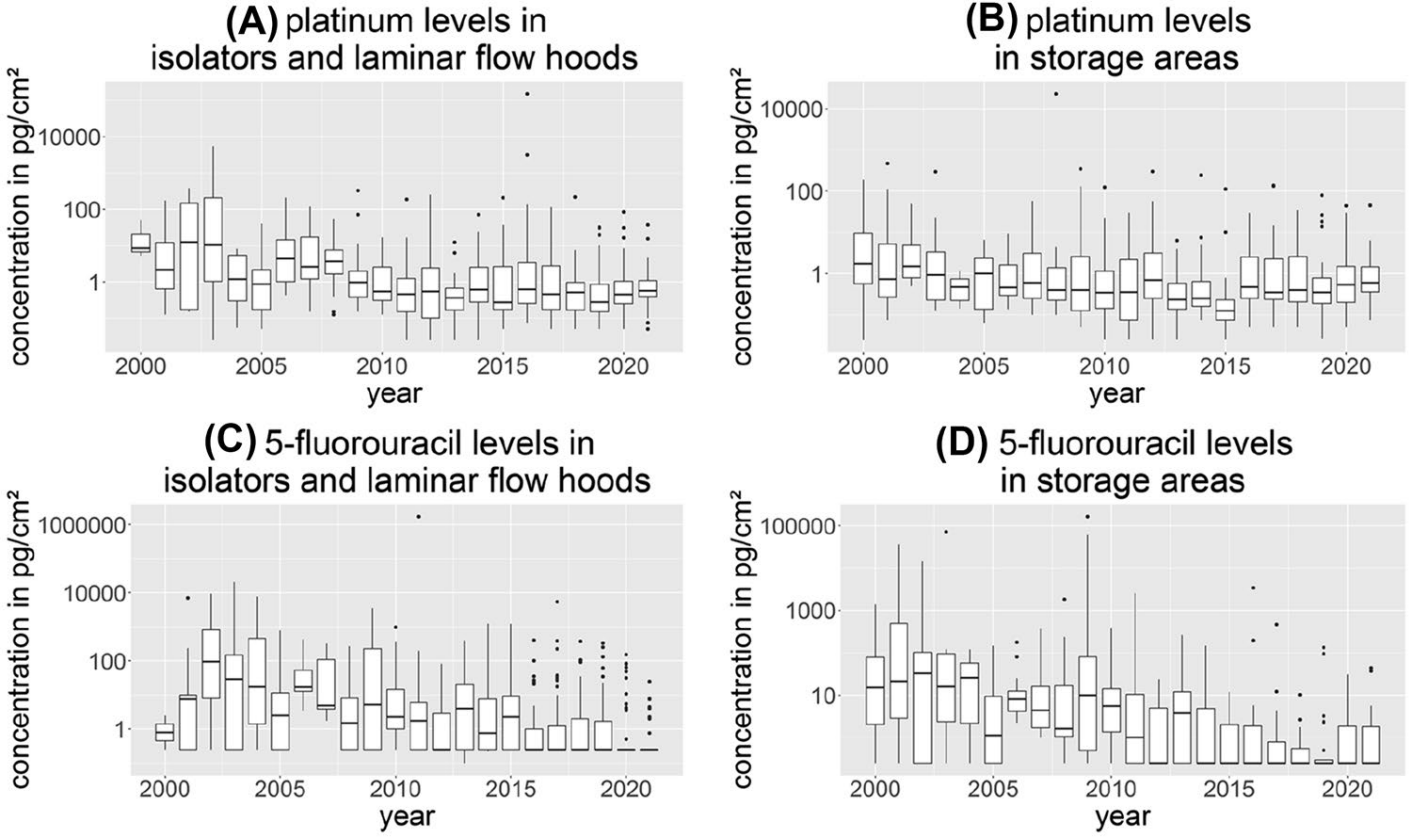
Development of surface contamination for platinum and 5-fluorouracil in laminar flow hoods and isolators (**A**, **C**) and storage areas (**B**, **D**)

The development of surface contamination in laminar flow hoods and isolators as well as storage areas is shown in Fig. 3 for Pt and FU. In both areas, surface contamination decreases with time. This trend was not observed of CP and GEM (Figure S3). Finally, multivariate linear regression models were performed to evaluate potential relationships between the outcomes and the factors. However, no additional information other than provided above was provided by these models. A table of the results can be found in the supplementary information.

## Discussion

The goal of this follow-up was (1) to evaluate the time trend of AD surface contaminations over time and to identify (2) the most frequently found AD as well as (3) the most critical locations in terms of surface contaminations and (4) to adjust the existing GV.

Overall, surface contaminations with Pt and FU decreased since the introduction of wipe sampling in pharmacies. This trend is particularly visible for FU and concentrations are still decreasing up to now. This can be explained by the increasing awareness of pharmacies and their efforts to improve the manufacturing of AD preparations, e.g. by introducing closed-system drug transfer devices (Harrison et al. 2006; Sessink et al. 2011; Soefje et al. 2022). This was also found in other international studies, (Chabut et al. 2021; Saint-Lorant et al. 2023; Sottani et al. 2010). However, in a recent study from Italy no clear trend in reduction of surface contamination was found for CP, FU, GEM and Pt between 2016 and 2021 (Sottani et al. 2022), which is in line with this study’s observations made for Pt, CP and GEM in the same period. In contrast, levels of FU decreased continuously.

Despite developments in anticancer therapy such as monoclonal antibodies, FU is still used in many treatment regimens. In fact, the reduction of surface contamination cannot be explained by lower usage. Studies in Canada demonstrated that FU, the AD with the highest reported use, is not the AD with highest detection rate in surface samples (Chabut et al. 2021; Delafoy et al. 2023). In contrast, other AD with lower usage such as CP and GEM were more frequently detected. This may be explained by different chemical properties that affect the stability of an AD and its adsorption to different surface materials. Consequently, some AD may not be efficiently removed by cleaning or detected by wipe sampling compared to others (Bláhová et al. 2021; Federici et al. 2019).

For Pt, the apparent stabilization of surface contaminations can be explained by the fact that the limit of detection is ten times lower compared to the other AD. Consequently, even low background concentrations can be determined. Furthermore, we analyzed total Pt as a proxy for Pt-based AD such as cisplatin. Therefore, Pt contaminations may not be caused by the primary AD but degradation products or not even related to AD processing at all, e.g. by urban dust. Nevertheless, Pt can be mainly attributed to AD processing as pharmacies work under clean room conditions, reducing outside sources of contamination to a minimum.

In contrast to FU and Pt, no trend was observed for most of the quantiles of the other AD or the concentrations were mainly below the LOD, respectively. This may partly due the limited stability of the AD, as our methods do not include potential degradation products. Furthermore, the used multimethods may be also applied in pharmacies or on surfaces where not all of the included AD were used, a priori eliminating the chance of a positive sample. However, we did not collect information on the quality and quantity of AD used from each pharmacy.

Data from our own working group (Schierl et al. 2016) as well as other groups demonstrated that the introduction of the monitoring programs lead to the reduction of surface contaminations (Chabut et al. 2021; Crul et al. 2020; Dugheri et al. 2018; Sottani et al. 2010). In order to identify current critical AD and locations, we focused on the data collected between 2015 and 2021. Similar to our study, Pt, CP and GEM were the AD with highest detection or exceedance rate in a recent study by Delafoy and colleagues (Delafoy et al. 2023). The high rate of exceedances for Pt in our study may be explained by the high amount of formulations used in chemotherapy and consequently a more frequent handling of Pt-based AD, like cisplatin, oxaliplatin or carboplatin. Furthermore, the GV for Pt are lower compared to the other AD despite a comparable molecular weight. In contrast to other AD, contaminations with Pt were much more likely to be in the intermediate range than in the high range. Although many surfaces were contaminated, it seems that either the spillage is relatively low or that Pt-based are more efficiently removed by cleaning procedures. Furthermore, there is no unified cleaning protocol for pharmacies. However, most pharmacies use a two-stage protocol including a polar solution (e.g. 0.05 M sodium hydroxide solution) followed by a more non-polar solution (e.g. 70% isopropanol) as recommended by Korczowska et al. (2020). This is very effective for the removal of most AD. Another possible explanation that should be mentioned is that the recovery rate for platinum compounds on the sampled surfaces may be low. A Dutch study was able to show that up to 13.3% of the original amount of platinum added was not recovered after desorption and analysis (Brouwers et al. 2007). The authors were able to show that in stainless steel, up to 49.6% of the original amount of platinum applied to the surface was lost. They attributed this not only to the inability of the wiping process used to absorb all of the added platinum but also to the variation in analysis and loss due to adsorption to the fabrics. For linoleum, up to 23.2% of the original amount of platinum applied to the surface was not recovered.

In comparison, FU exceedance rates were much lower. This can be explained by the relatively high GV proposed for FU in 2009 (Schierl et al. 2009). Since the concentrations for FU decreased considerably, lower exceedance rates were not surprising. Furthermore, even though the quantiles CP and GEM were lower, exceedance rates were higher compared to FU. Here, a lower GV for CP and GEM was the reason for this finding. As mentioned above, the low exceedance rates for MTX, DOC and PAC may be a result of their inclusion in the multimethod.

High exceedance rates for laminar flow hood and the isolator were expected as the handling of AD solutions is carried out mainly at these locations. This finding is consistent with the results of other studies, which also showed that the laminar flow hoods, the floor in front of the hoods, the door handles, and the maintenance hatch handles were frequently contaminated (Brouwers et al. 2007; Delafoy et al. 2023; Korczowska et al. 2020). Spillage can occur during the opening of bottles, pipetting solutions or due to residual amounts of AD on the used materials. It is suspected that this is due to improper use of work procedures or inadequate cleaning. In storage locations, residual amounts of ADs on newly ordered or already opened stock solutions are likely the major cause of contaminations (Silva et al. 2003.; Hilliquin and Bussières 2020). Nevertheless, contamination in laminar flow hoods, isolators and storage areas decreased over time. This is probably due to the increased awareness and improved handling procedures by the pharmacies such as using close-system drug transfer devices, single-use sheets in storage areas or cleaning of AD vials after receipt.

Floors, in particular linoleum floors, are a good absorbing material for many AD, especially non-polar substances. Consequently, contaminations are likely to occur as the spillage may have happened weeks or even months prior to the sampling. This possibility was already considered by Chabut et al. (Chabut et al. 2021). We noticed that even after vigorous cleaning, contaminations with AD were still detectable. Surprisingly, even non-contact areas such as door handles and keyboards frequently exceeded GV. This may be a result of cross-contamination, e.g. by contaminated gloves. These results are consistent with the results of other studies (Brouwers et al. 2007; Chabut et al. 2021).

Despite of cleaning and safety procedures in place, there is still a risk of exposure for pharmacy staff and pharmacies are obliged to keep surface contamination as low as possible. Consequently, the GV that we currently use to evaluate wipe samples must be adjusted to (1) account for the general reduction of contamination since 2009 and (2) to provide incentives to even further reduce surface contamination. For the determination of adjusted GV, the data from 2015 to 2021 have been used. In 2009, Schierl et al. used the P50 and P75 for GV-I and GV-II, respectively, for FU and Pt (Schierl et al. 2009). However, the P50 for all AD other than Pt were already below the LOD and we, therefore, decided to use the LOD as adjusted GV-I. Consequently, a result above the LOD must be considered as a preventable contamination. Since the majority of pharmacies was able to achieve results below the LOD, all others should intend to do the same. For Pt, the derivation of GV-I also accounts for background levels. If possible, P90 was used for the determination of GV-II. The P90 was also used by Delafoy and colleagues as a guidance value (Delafoy et al. 2023). If the P90 is below the LOD, we propose to use tenfold the LOD as GV-II. A summary of the adjusted GV can be found in Table 4. Using the adjusted GV, pharmacies are able to compare their individual results and optimize their working procedures according to the current state of quality and risk management. We are aware that our GV depend on the sensitivity of the analytical method and cannot be averted internationally to all corresponding workplaces in pharmacies due to the different workplace situations. Nevertheless, from our point of view, they provide valuable reference points for reviewing the own work procedures and comparing them with empirical values used over many years.

**Table 4:** Proposal of adjusted guidance values (GV) for antineoplastic drugs (AD) based on the data between 2015 and 2021

AD	GV-I in pg/cm^2^	GV-II in pg/cm^2^
Pt	0.6	2.6
FU	0.5	2.7
CP	0.5	3.8
IF	0.5	5.0
GEM	0.5	3.5
MTX	0.5	5.0
DOC	0.5	5.0
PAC	0.5	5.0

It should be considered that contaminations with small-molecular AD, as analyzed in our study, are likely to be influenced by the improvements in anticancer therapy, e.g. by the increasing use of monoclonal antibody-based therapy methods. For the risk assessment of monoclonal antibodies (mAbs) the following two aspects are of central importance: the toxicological properties of the active substances or the preparations and the risk of harmful exposure. Although they have been in clinical use for more than 30 years, the conditions for occupational safety for working with mAbs have not yet been described. The assessment of the individual substance properties of mAb and the assessment of the relevance for the health of employees in the health service are currently only possible to a limited extent due to the poor data situation (Gerding 2018). In view of the widespread use of mAbs, future studies should focus on the detection of workplace surface contamination with mAbs to detect the workplace surface contamination and to implement sufficient protective measurements.

## Limitations of the study

This study only includes basic information on the pharmacies and the analytical results. For a more detailed interpretation of the results, information on amounts of AD handled, cleaning processes, compounding techniques and other organizational features would have been required. However, the presented data were acquired in the course of a service to pharmacies and not within a scientific study.

## Conclusion

The results showed that surface contaminations with AD decreased significantly over the past two decades and are now at a relatively low level. Furthermore, occupational exposure is reduced to a minimum due the use of personal protective equipment. Nevertheless, wipe sampling is still a useful tool for quality and risk management in terms of validation of cleaning validation and occupational health. Although many more AD than the substances presented in this study are used in chemotherapy, it provides a good insight into the handling of AD in pharmacies and the risk of surface contamination.

## Supplementary Information

Below is the link to the electronic supplementary material.

Supplementary file1 (PDF 842 KB)

Supplementary file2 (XLSX 18 KB)

## Data Availability

The data can be made available upon reasonable request.
