# Supplementary material for: Evaluation of long-term data on surface contamination by antineoplastic drugs in pharmacies
Source: Int Arch Occup Environ Health. 2023 Mar 6;96(5):675–83. doi: 10.1007/s00420-023-01963-y (PMC10219872; doi:10.1007/s00420-023-01963-y)

Supplementary Information

Analytical Methods

Total platinum (Pt): For analysis, the wipe samples were extracted with 25 ml 2 % hydrochloric acid for one hour and analyzed by voltammetry. One milliliter of the extract was transferred into a quartz vessel and 5 ml ultrapure water, 100 µl sulfuric acid (96 %) and 200 µl hydrogen peroxide (30 %) were added. The samples were UV-irradiated for 2 h using a MAUV-2X UV digestor (Maassen, Reutlingen, Germany) and afterwards analyzed by voltammetry for Pt determination (Methrom, Filderstadt, Germany). The quantitation based on the standard addition method. In detail, between 10 and 100 pg of platinum were added directly to the measuring solution after initial the voltammetric analysis. The limit of detection per sample was 0.05 ng/sample.

5-Fluorouracil (FU): FU concentrations were quantified by gas chromatography-tandem mass spectrometry (GC-MSMS). The samples were extracted with 30 mL of methanol was added and samples were placed on a shaker for 30 min at 180 rpm. Aliquots of 10 mL were transferred into a glass tube and spiked with internal standard (IS) solution (FU-$^{13}C_3$) and the solvent was evaporated with nitrogen. Prior to injection, FU was derivatized using BTFSA. GC-MS/MS analysis was performed with a system from Agilent Technologies (Waldbronn, Germany) composed of a 7890A GC coupled to a 7000 GC-MS triple quad. The analytical system included a VF-5ms chromatography capillary column (15 m × 0.25 mm inner diameter; 0.25 µm film thickness), and the constant flow of carrier gas (He) was 1.5 mL/min. The MS parameters were optimized for maximum sensitivity. An external calibration was performed in the range from 0.01 to 2.5 ng/ml. Samples with higher concentrations were diluted accordingly.

Cyclophosphamide (CP) and ifosfamide (IF) by GC-MS/MS: Wipe samples for only CP and IF were analyzed GC-MSMS. The samples were extracted with 30 mL of ethyl acetate and the samples were placed on a shaker for 30 min at 180 rpm. Aliquots of 10 mL were transferred into a glass tube and spiked with IS (CP-d_6) and the solvent was evaporated with nitrogen. Prior to injection, CP and IF were derivatized using acetic anhydride. GC-MS/MS analysis were performed with a system from Agilent Technologies (Waldbronn, Germany) composed of a 7890A GC coupled to a 7000 GC-MS triple quad. The analytical system included a VF-5ms chromatography capillary column (15 m × 0.25 mm inner diameter; 0.25 µm film thickness), and the constant flow of carrier gas (He) was 1.5 mL/min. The MS parameters were optimized for maximum sensitivity. An external calibration was performed in the range from 0.01 to 2.5 ng/ml. Samples with higher concentrations were diluted accordingly.

Cyclophosphamide (CP), ifosfamide (IF), gemcitabine (GEM), methotrexate (MTX), docetaxel (DOC), paclitaxel (PAC) by LC-MS/MS: The samples were extracted with 30 mL of methanol and the samples were placed on a shaker for 30 min at 180 rpm. Aliquots of 10 mL were transferred into a glass tube and spiked with IS (CP-d_6, GEM-$^{13}C^{15}N_2$, MTX-d_3, DOC-d_9, PAC-d_5) and the solvent was evaporated with nitrogen. Prior to injection, samples were dissolved in 100 µl methanol. LC-MS/MS analysis were performed with a system composed of an Agilent Technologies 1260 HPLC (Waldbronn, Germany) and a Sciex 4000 MS/MS System (Framingham, MA, USA). Separation was achieved on a Kinetex® C18 HPLC column (150 x 2.1 mm, 2.7 µm, 100 Å, Phenomenex, Torrance, CA, USA) at a flow rate of 0.3 ml/min. 0.1 % formic acid in water and 0.1 % formic acid in a mixture of methanol and isopropanol (1:1) were used as mobile phases. The MS parameters were optimized for maximum sensitivity. An external calibration was performed in the range from 0.01 to 2.5 ng/ml. Samples with higher concentrations were diluted accordingly.

Table S1: Detailed description of the sampling locations in pharmacies.

location	description
laminar flow hood	processing of antineoplatic drug (AD) base solutions, preparation of infusion solutions
isolator	similar to a laminar flow hood but with a closed front window
preparation area	unpacking of AD base solutions and other incoming goods
post-processing	packing and sealing of finished infusion solutions
floor laminar flow hood	floor in front of the laminar flow hood
other floors	floor in front of storage, disposal areas, shelves, refridgerators, etc.
material lock	material and document locks, transfer hatch
transport box	transports boxes for AD base solutions and infusion solutions
pactosafe	device for safe AD disposal
storage	shelves, cupboard, refridgerators with opened and unopened AD base solutions
indirect contact areas	locations without direct AD processing/contact, e.g. door handles, keyboards, telephones, desks, etc.

Table S2: Exceedances of guidance values (GV) for individual ADs from 2015 to 2021.

AD	number of individual results	GV-I < n < GV-II (%)	n > GV-II (%)	total n > GV (%)
Pt	2,223	436 (19.6)	163 (7.3)	599 (26.9)
FU	2,722	131 (4.8)	74 (2.7)	205 (7.5)
CP	1,589	157 (9.9)	137 (8.6)	294 (18.5)
IF	1,589	51 (3.2)	75 (4.7)	126 (7.9)
GEM	1,343	116 (8.6)	108 (8.0)	224 (16.7)
MTX	1,342	11 (0.8)	3 (0.2)	14 (1.0)
DOC	1,342	19 (1.4)	8 (0.6)	27 (2.0)
PAC	1,343	27 (2.0)	16 (1.2)	43 (3.2)
**Total**	**13,493**	**948 (7.0)**	**584 (4.3)**	**1532 (11.4)**

Table S3: Exceedances of guidance values (GV) for all ADs at individual sampling locations from 2015 to 2021.

location	number of individual results	GV-I < n < GV-II (%)	n > GV-II (%)	total n > GV (%)
laminar flow hood	2,569	227 (8.8)	200 (7.8)	427 (16.6)
isolator	319	47 (14.7)	31 (9.7)	78 (24.5)
preparation area	2,179	115 (5.3)	33 1.5)	148 (6.8)
post-processing	1,349	60 (4.4)	38 (2.8)	98 (7.3)
floor laminar flow hood	1,018	111 (10.9)	52 (5.1)	163 (16.0)
other floors	824	71 (8.6)	33 (4.0)	104 (12.6)
material lock	795	37 (4.7)	10 (1.3)	47 (5.9)
transport box	550	19 (3.5)	8 (1.5)	27 (4.9)
pactosafe	320	8 (2.5)	10 (3.1)	18 (5.6)
storage	1,264	121 (9.6)	101 (8.0)	222 17.6)
indirect contact areas	2,229	131 (5.9)	67 (3.0)	198 (8.9)
**total**	**13,416**	**947 (7.1)**	**583 (4.3)**	**1530 (11.4)**

Figure S1: Development of selected percentiles (P50, P75, P90, P95) of surface contaminations with ifosfamide (A, IF), methotrexate (B, MTX), docetaxel (C, DOC) and paclitaxel (D, PAC) between 2000 and 2021.

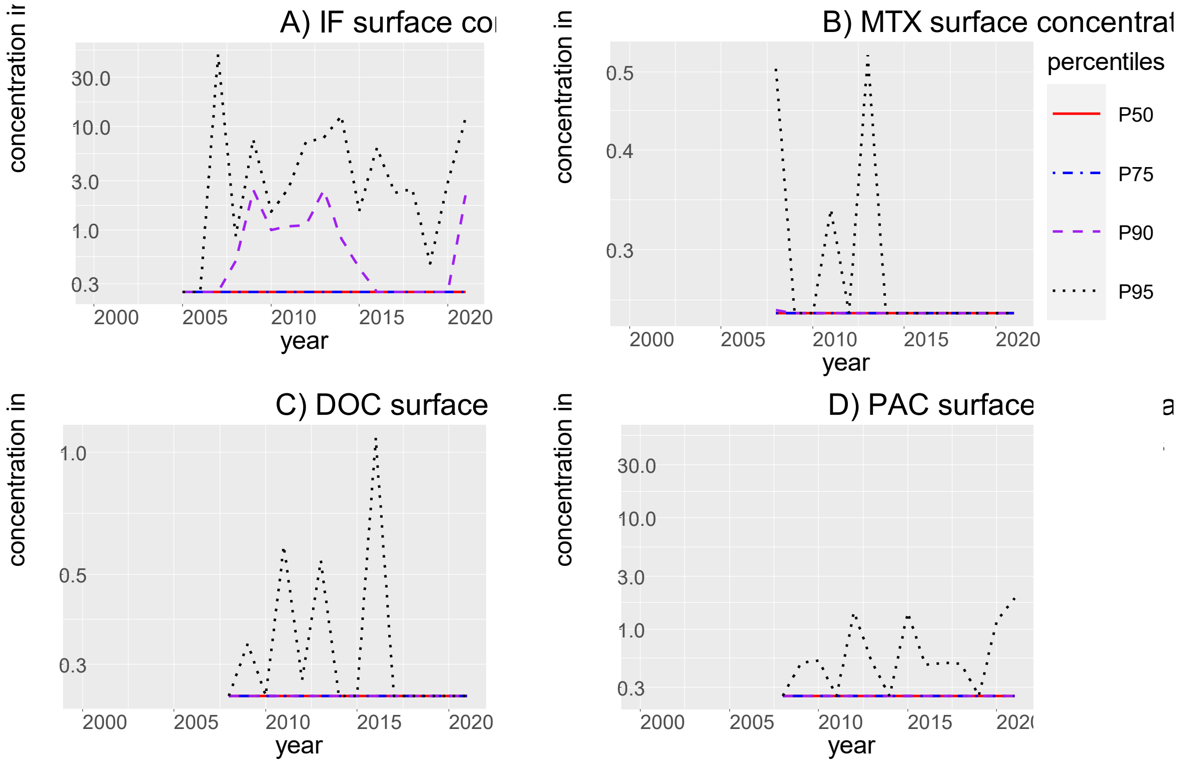

Figure S2: Surface contamination of platinum (A), 5-fluorouracil (B), cyclophosphamide (C) and gemcitabine (D) in relation to times participated in the wipe sampling program.

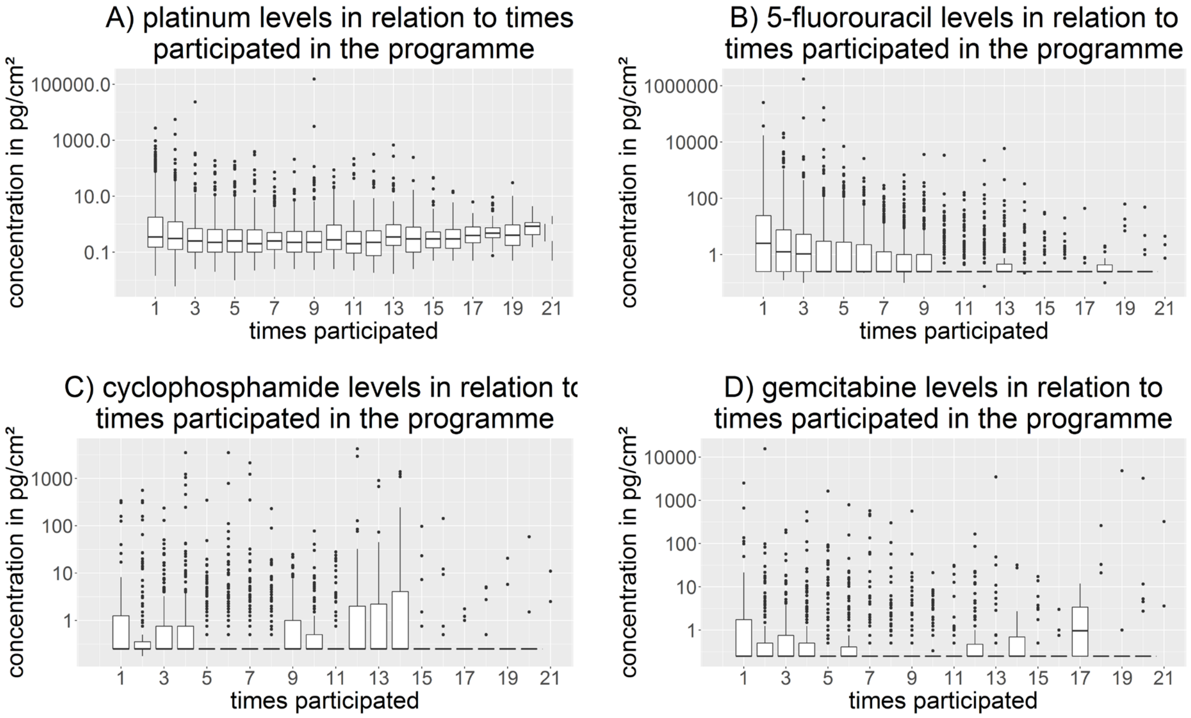

Figure S3: Development of surface contamination for cyclophosphamide and gemcitabine in laminar flow hoods and isolators (A, C) and storage areas (B, D).

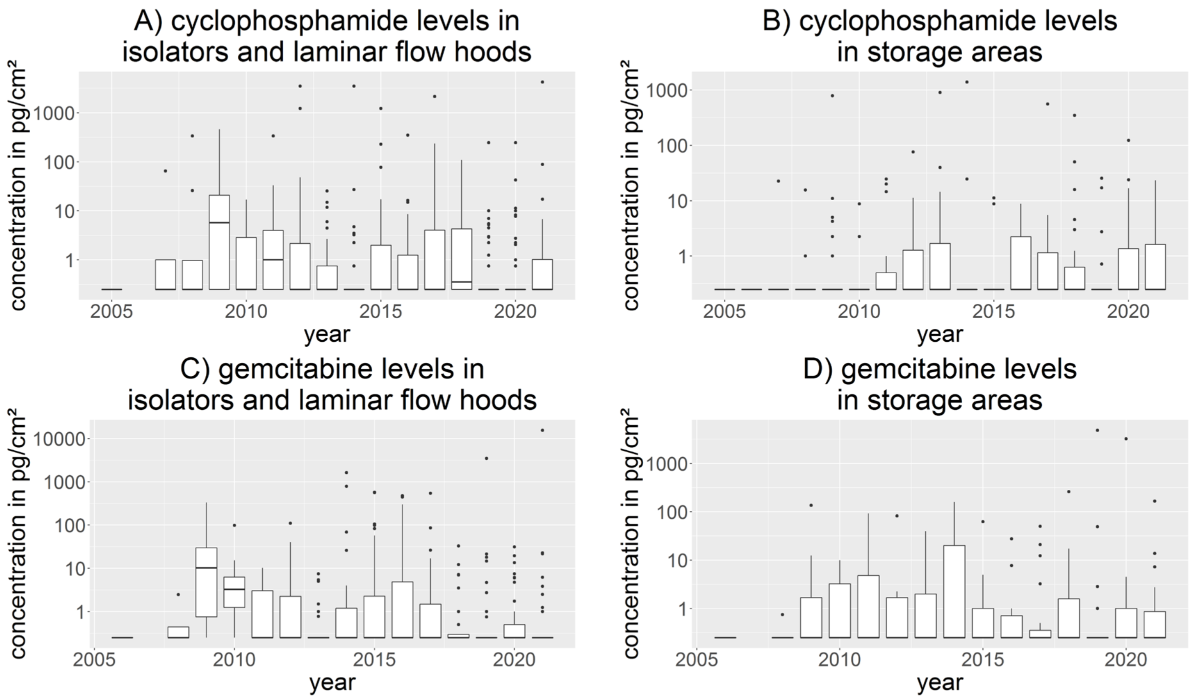

Supplement: Supplementary file 1 — Supplementary file1 (PDF 842 KB) [file 420_2023_1963_MOESM1_ESM.pdf]
